# *In vitro* anti-*Leishmania* activity of triclabendazole and its synergic effect with amphotericin B

**DOI:** 10.3389/fcimb.2022.1044665

**Published:** 2023-01-09

**Authors:** Beatriz Santana Borges, Gislayne de Paula Bueno, Fernanda Tomiotto-Pellissier, Fabiano Borges Figueiredo, Lia Carolina Soares Medeiros

**Affiliations:** ^1^ Instituto Carlos Chagas, Fundação Oswaldo Cruz (Fiocruz), Curitiba, Paraná, Brazil; ^2^ Laboratory of Immunopathology of Neglected Diseases and Cancer (LIDNC), Department of Pathological Sciences, State University of Londrina, Londrina, Paraná, Brazil; ^3^ Department of Medical Pathology, Federal University of Paraná, Curitiba, Paraná, Brazil

**Keywords:** triclabendazole, drug repurposing, Leishmaniasis, benzimidazoles, *Leishmania amazonensis*

## Abstract

**Introduction:**

Leishmaniasis is a neglected tropical disease, with approximately 1 million new cases and 30,000 deaths reported every year worldwide. Given the lack of adequate medication for treating leishmaniasis, drug repositioning is essential to save time and money when searching for new therapeutic approaches. This is particularly important given leishmaniasis’s status as a neglected disease. Available treatments are still far from being fully effective for treating the different clinical forms of the disease. They are also administered parenterally, making it challenging to ensure complete treatment, and they are extremely toxic, in some cases, causing death. Triclabendazole (TCBZ) is a benzimidazole used to treat fasciolosis in adults and children. It presents a lower toxicity profile than amphotericin B (AmpB) and is administered orally, making it an attractive candidate for treating other parasitoses. The mechanism of action for TCBZ is not yet well understood, although microtubules or polyamines could potentially act as a pharmacological target. TCBZ has already shown antiproliferative activity against *T. cruzi, T. brucei*, and *L. infantum*. However, further investigations are still necessary to elucidate the mechanisms of action of TCBZ.

**Methods:**

Cytotoxicity assay was performed by MTT assay. Cell inhibition (CI) values were obtained according to the equation CI = (O.D treatment x 100/O.D. negative control). For Infection evaluation, fixated cells were stained with Hoechst and read at Operetta High Content Imaging System (Perkin Elmer). For growth curves, cell culture absorbance was measured daily at 600 nm. For the synergism effect, Fractional Inhibitory Concentrations (FICs) were calculated for the IC50 of the drugs alone or combined. Mitochondrial membrane potential (DYm), cell cycle, and cell death analysis were evaluated by flow cytometry. Reactive oxygen species (ROS) and lipid quantification were also determined by fluorimetry. Treated parasites morphology and ultrastructure were analyzed by electron microscopy.

**Results:**

The selectivity index (SI = CC50/IC50) of TCBZ was comparable with AmpB in promastigotes and amastigotes of *Leishmania amazonensis*. Evaluation of the cell cycle showed an increase of up to 13% of cells concentrated in S and G2, and morphological analysis with scanning electron microscopy showed a high frequency of dividing cells. The ultrastructural analysis demonstrated large cytoplasmic lipid accumulation, which could suggest alterations in lipid metabolism. Combined administration of TCBZ and AmpB demonstrated a synergistic effect *in vitro* against intracellular amastigote forms with cSFICs of 0.25.

**Conclusions:**

Considering that TCBZ has the advantage of being inexpensive and administrated orally, our results suggest that TCBZ, combined with AmpB, is a promising candidate for treating leishmaniasis with reduced toxicity.

## 1 Introduction

Leishmaniasis is a neglected disease caused by at least 18 protozoan species of the genus *Leishmania*, leading to up to 1 million cases per year worldwide (Burza et al., 2018). The disease presents three clinical forms, including cutaneous and mucocutaneous forms, which can cause permanent skin scarring, and the visceral form, which leads to death in 90% of the cases if not treated, being the parasitic disease with the second highest mortality index (Pace, 2014).

Despite being less severe than the visceral form, cutaneous leishmaniasis frequently leads to a large degree of psychological suffering, social stigma, and isolation due to the lesions, which, although usually self-healing, can be a source of secondary infections if left untreated (Pires et al., 2019). Approximately 300,000 cases per year of cutaneous and diffuse cutaneous clinical forms in South America are caused by *Leishmania amazonensis* (Burza et al., 2018).

Available treatments of all clinical forms are toxic, expensive, and/or difficult to administrate and cause severe side effects. Meglumine antimoniate and amphotericin B (AmpB) are the most common treatments (first- and second-line drugs, respectively), and both require prolonged intravenous administration, making the patient less likely to comply with the treatment fully. In its liposomal formulation, AmpB has been indicated for patients after the failure of the first-line treatment, for those with severe disease, those presenting comorbidities or immunodeficiencies, and for pregnant women (Croft and Olliaro, 2011). Pentamidines, paromomycin and miltefosine (first orally administered drug for leishmaniasis) can also be used in endemic areas, despite also having a high level of toxicity and therapeutic failure (Santi and Murta, 2022).

The development of new drugs is a long and expensive process. Successful treatments should have a specific pathogen target in order to eliminate the pathogen without harming the host (Ioset et al., 2009). Due to the high costs involved in treating parasitic diseases, they are often neglected by pharmaceutical companies.

Drug repositioning is a quicker way of providing new compounds for a wide range of diseases for which no adequate treatment currently exists. These drugs have already been approved by US Food and Administration (FDA), saving a great amount of time and money, and their safety profile is well-known (Nosengo, 2016). A variety of repositioning drugs have been frequently reported for treating conditions, including bipolar disorder, migraines, type 2 diabetes and pulmonary hypertension (Alberca et al., 2016). Also, tropical neglected diseases present a range of repurposed drugs either from other approved treatments or veterinary use such as eflornithine for Human African Trypanosomiasis (HAT) and albendazole for lymphatic filariasis (Klug et al., 2016). Indeed, some drugs currently used for leishmaniasis treatment are repurposed, such as amphotericin B, paromomycin and miltefosine, originally used as antifungal, antibiotic and antineoplastic, respectively (Field et al., 2017). In this context, it is important to highlight the benzimidazole family of drugs, which has not only demonstrated a satisfactory anti-parasitic effect but has also been shown to have good patient tolerability in a variety of benzimidazole derivates (Bansal and Silakari, 2012).

One member of the benzimidazole family is triclabendazole (6-chloro-5-(2,3-dichloro phenoxy)-2-(methylthio)-1H-benzimidazole) (TCBZ), a drug that has been used to treat fascioliasis in animals since the early 1980s and which was approved for adult use in the early 1990s. It was also approved for infantile fascioliasis (over six years) in 2019 (Terashima et al., 2021). TCBZ has already demonstrated a bactericidal effect as a successful repurposed drug (Pi et al., 2021), and antiproliferative *in vitro* activity against *Trypanosoma cruzi* (Alberca et al., 2016). Further experiments have not yet been performed to investigate the drug’s mechanism of action and its inhibitory effects on *Leishmania* spp.

TCBZ’s mechanism of action is still not properly understood, and it may target microtubules and/or protein synthesis (Robinson et al., 2002). Still, it appears to be a very safe drug. TCBZ has shown a well-tolerability, with mild side effects such as biliary colic and abdominal pain, which is consistent with the expulsion of dead flukes parasites (*Fasciola hepatica*) (Gandhi et al., 2019). TCBZ also has the advantage of being administered orally in one, two, or three doses, facilitating the continuation and conclusion of the patient’s treatment (Terashima et al., 2021). Studies have shown that TCBZ absorption after oral administration is fast, with *t*_max_ (time to reach the maximum serum concentration) of 2 to 3 hours (Gandhi et al., 2019). In addition, treatment with liposomal AmpB costs over 1,200 USD for an individual weighing 70 kg (Datta et al., 2020), while treatment with TCBZ costs 3 to 5 USD for the same individual.

Given the current availability of treatments for leishmaniasis and the importance of drug repositioning, especially for neglected tropical diseases, we tested the anti-*Leishmania* activity of TCBZ against the *L. amazonensis* strain alone and in combination with AmpB.

## 2 Material and methods

### 2.1 In silico study

The AmpB and TCZB structures were used to evaluate their theoretical physicochemical properties, whether PAINS are present, and the drug interaction prediction. The predictions were calculated using the swissADME web tool (http://www.swissadme.ch) considering Lipinski’s rule of five (Ro5) (Lipinski, 2004) followed by the additional rule proposed by Veber (Veber et al., 2002) and PAINS presence (Baell and Holloway, 2010). Drug interaction prediction was performed using the Drug Interaction Checker of DrugBank (https://go.drugbank.com/drug-interaction-checker).

### 2.2 Parasites

*Leishmania amazonensis* (strain MHOM/BR/73/M2269) promastigotes were kept at 25°C in M199 medium added with 40 mM HEPES, 26 mM NaOH, 5 µg/mL of hemin supplemented with 10% of fetal bovine serum (FBS, Gibco^®^). The parasites were cultivated for three days for logarithm growth stage parasite assays and seven days for stationary growth stage parasites assays.

### 2.3 Cytotoxicity assay

Cytotoxicity assay was performed as previously described (Ceole et al., 2017). Cell viability was evaluated by MTT (3-(4,5-dimethylthiazol-2-yl)-2,5-diphenyltetrazolium bromide) assay. Optical density (O.D.) was read in an ELISA plate reader (Biotek Model EL-800, VT, USA) at 570 nm. In order to calculate the 50% cytotoxic concentration index (CC_50_), THP-1 (ATCC TIB-202) cells were seeded in 96-well plates at density of 25x10^4^ cells/mL in RPMI 1640 medium containing 50 ng/mL of PMA (Phorbol 12-myristate 13-acetate) for 48h as previously described (Alcântara et al., 2020). The plates were incubated at 37°C in a humidified incubator with an atmosphere of 5% CO_2_ to induce differentiation into adherent macrophages. After this, macrophages were incubated with increasing concentrations of TCBZ or AmpB for 48 or 72h in a CO_2_ incubator. Concentrations used for AmpB ranged from 0 to 10 μM (0, 0.009, 0.019, 0.039, 0.078, 0.156, 0.312, 0.625, 1.25, 2.5, 5 and 10 μM), while concentrations used for TCBZ ranged from 0 to 100 μM (0, 0.097, 0.195, 0.39, 0.78, 1.56, 3.125, 6.25, 12.5, 25, 50 and 100 μM). Then, 50 µL of MTT at 5 mg/mL was added per well, and the plates were incubated for 4h at 37°C. The plates had their medium removed, and 25 µL of 10% SDS solution in HCL was added for cell lysis. Subsequently, 50 µl of DMSO was added for the elution of formazan crystals. The colorimetric assay was read using optical density at 570 nm. Cell inhibition (CI) values were obtained from three experiments performed in triplicate according to the equation CI = (O.D treatment x 100/O.D. negative control) (Escobar et al., 2010; Ceole et al., 2017).

### 2.4 Infection evaluation

Infection assays were performed as previously described (Alcântara et al., 2020). Differentiated macrophages (25x10^4^ cells/mL) were infected with metacyclic promastigotes (5 to 7-days-old metacyclic promastigote cells culture) at a ratio of 50:1 in RPMI media for 24h using 96 well-plates placed in a humidified incubator at 34°C with an atmosphere of 5% CO_2._ Previously to cells treatment, infection tests were made in three different time-points (24, 48 and 72h) for determination of higher infection index.

For cells treatment, increasing concentrations of AmpB and TCBZ were added to cell media and incubated for 48h at 34°C and 5% CO_2_ after infection. Concentrations used for AmpB ranged from 0 to 20 μM (0, 0.009, 0.019, 0.039, 0.078, 0.156, 0.312, 0.625, 1.25, 2.5, 5 and 10 μM), while concentrations used for TCBZ ranged from 0 to 500 μM (0, 0.97, 1.95, 3.9, 7.8, 15.6, 31.25, 62.5, 125, 250 and 500 μM). Plates were washed twice with PBS, and cells were fixated with 4% PFA for 20 min, washed twice with ultrapure H_2_O, stained with Hoechst for 20 min, and read at Operetta High Content Imaging System (Perkin Elmer). The number of intracellular amastigotes was evaluated to calculate half-maximum inhibitory concentration (IC_50_). Non-treated wells were considered as having 100% viability of amastigotes.

### 2.5 Antiparasitic evaluation

Growth curve was performed using three-day-old promastigote cells culture of *L. amazonensis.* Parasites were harvested and had their concentration adjusted to 1x10^6^ parasites/mL and seeded in 24 well plates in 1.5 mL of M199 media. Non-treated parasites were used as control. Promastigotes were treated with increasing concentrations of AmpB (0.312, 0.625, 1.25, 2.5 and 5 μM) or TCBZ (3.125, 6.25, 12.5, 25 and 50 μM) and maintained in a BOD incubator at 25°C for 72h. Plates had absorbance measured daily for drug effect analysis at 600 nm (Kar et al., 2021).

For determination of promastigotes IC_50,_ 3-day-old culture of promastigotes was seeded in 96 well plates at a concentration of 10^6^ parasites/mL in the presence of AmpB (0, 0.019, 0.039, 0.078, 0.156, 0.312, 0.625, 1.25 and 2.5 μM) or TCBZ (0, 0.097, 0.195, 0.39, 0.78, 1.56, 3.125, 6.25, 12.5, 25, 50 and 100 μM) in M199 media. Plates were maintained at 25°C for 72h. After incubation, promastigotes viability was accessed by MTT assay (as described at item 2c).

### 2.6 Synergism of TCBZ and AmpB

Infected macrophages were treated with TCBZ and AmpB using a ratio of 10:1, 5:1, 2:1, and 1:1 in five serial dilutions (base 2) for 48h. The molar concentration for each ratio was designed as follows: 40 μM:4μM; 20μM:4 μM; 16μM:4μM and 4μM:4μM for TCBZ and AmpB respectively After the plates were analyzed using the Operetta CLS High Content Analysis System, Fractional Inhibitory Concentrations (FICs) were calculated for the IC_50_ of the drug alone and combined (Trinconi et al., 2014; Kar et al., 2021). Isobolograms were plotted using the sum of FICs (ΣFICs) of the ratio of each combined drug. The average of FICs sum of all ratios (χΣFICs) was calculated and the combinatory effect was determined according to Odds (2003), where χΣFICs ≤ 0,5 is synergic, χΣFICs > 0,5< 4 is indifferent, and χΣFICs > 4 is antagonist. The equation for the ΣFIC_50_ calculation is described below:


$$\text{ΣFICs}=\;\left[\;\frac{\;IC_{50}\;\text{drug A in combination}}{IC_{50}\;\text{drug A alone}}\;\right]\;+\;\left[\;\frac{\;IC_{50}\;\text{drug B in combination}\;}{IC_{50}\;\text{drug B alone}}\;\right]$$

To the present, there is no gold standard method established to test antimicrobial synergism. Although the method used in this work can be limited to only testing antimicrobials for a fixed incubation time, it was used because it is a well-established method.

### 2.7 Flow cytometry

*Leishmania amazonensis* (10^6^ promastigotes/mL) were previously treated with TCBZ (IC_50_ for 72h). To assess the mitochondrial membrane potential (ΔΨm), the promastigotes were washed with PBS and incubated for 15 min at 26°C with 5 μg/mL of rhodamine 123 (Rh123) and immediately evaluated in a flow cytometer. For cell cycle analysis, promastigotes were resuspended in 500 μL of PBS, to which the same volume of permeabilizing solution and DNA staining solution was added (3.4 mM Tris-HCl, pH 7.4; 0.1% NP-40; 700 U/mL free RNase A-DNase, 10 mM NaCl and 75 μM propidium iodide). A fluorescence reading was taken after an incubation period of 10 min at room temperature. To evaluate cell death, promastigotes were resuspended in annexin binding buffer (140 mM NaCl, 5 mM CaCl_2,_ and 10 mM HEPES-Na, pH 7.4) and labeled with Annexin V conjugated to Alexa Fluor 488 and 100 μg/mL of propidium iodide (PI). In all cases, events were read in a FACSCanto II flow cytometer (Becton – Dickinson, San Jose, CA, USA), and the data were analyzed using FlowJo v10 (Treestar Software, Ashland, OR, USA).

### 2.8 Reactive oxygen species

ROS quantification was performed as described by Bortoleti et al. (Bortoleti BT da et al., 2021). Promastigotes were cultured in 1 mL of M199 in presence of AmpB or TCBZ IC_50_ for 72h. In positive controls, 0.2 µL of 2 mM H_2_O_2_ was added to 1 mL of cell solution 60 min before withdrawing the treatment. The parasites were collected through centrifugation (2000 rpm for 7 min), the supernatant was discarded, and the pellet was resuspended in 500 µL of M199. Parasites were plated in black 96-well plates (99 µL per well). An initial reading was performed to assess cellular autofluorescence in a fluorimeter (Ex 495; Em 520). Subsequently, 1 µL of the H_2_DCFDA solution was added (final concentration in the well of 20 µM). Plates were incubated for 1h in a 37°C incubator with an atmosphere of 5% CO_2,_ and, after this time, the fluorescence was read in a fluorimeter.

### 2.9 Lipid quantification

Neutral lipids were detected as described in Bortoleti et al. (Bortoleti BT da et al., 2021). Promastigote forms previously treated with TCBZ or AmpB (IC_50_) for 72h were collected and washed twice with PBS. After that, 199.5 µL of M199 and 0.5 µL of Nile Red solution (1 mg/mL) were added and incubated for 30 min in a BOD. Cells were rewashed and resuspended in 500 µL PBS. Then, the cell suspension was plated in black 96-well plates (100 µL per well), and the fluorescence reading was performed in a fluorimeter (Ex 530; Em 635).

### 2.10 Electron microscopy

Parasites were treated with TCBZ or AmpB (CI_50_) for 72h. The parasites were then harvested at a concentration of 1x10^6^ parasites/mL, washed in PBS, and fixed with Karnovsky fixation solution (2.5% glutaraldehyde, 4% paraformaldehyde in 0.1 M sodium cacodylate buffer) for 1h and washed with cacodylate buffer after incubation. Untreated parasites were also fixed and used as control. For scanning electron microscopy (SEM), cells were previously seeded in poly-L-lysin-coated coverslips. Cells were post-fixated with 1% osmium tetroxide for 1h in the dark and then dehydrated at growing ethanol concentrations (30 to 100%). Coverslips containing treated or untreated cells were submitted to critical point drying and then coated with a 20-nm-thick gold layer and observed under a JEOL JSM 6010 PLUS-LA (Akishima, Tokyo, Japan) scanning electron microscope. For transmission electron microscopy (TEM), treated and untreated cells were post-fixated with 1% osmium tetroxide for 1h in the dark, dehydrated using growing concentrations of acetone (30 to 100%) and infiltrated with increasing concentrations of Poly/Bed 812 resin and polymerized for 72h at 60°C. Ultrathin sections were contrasted for 30 min with uranyl acetate and 2 min with lead citrate. The ultrathin sections were observed in a JEOL JEM-1400 Plus transmission electron microscope operating at 80 kV.

### 2.11 Statistical analysis

Statistical analysis was performed in GraphPad Prism 8 (GraphPad Software, Inc. San Diego, CA, USA) using one or two-way ANOVA test followed by Dunnett’s post-test. IC_50_ assay results were transformed in logarithm values and analyzed by dose-response inhibition (* p-value<0.04; ** p-value<0.009; *** p-value<0.0009; **** p-value<0.00009). Results were plotted, showing the standard error of the mean. All experiments were performed in biological triplicate.

## 3 Results

### 3.1 In silico predictions for AmpB and TCBZ

**Figure 1:**
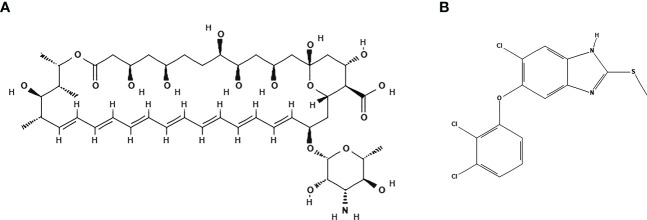
Structures of Amphotericin B and Triclabendazole. **(A)** Amphotericin B presents a molecular formula of C_47_H_73_NO_17_ (PubChem CID: 5280965). **(B)** Triclabendazole presents a molecular formula of C_14_H_9_C_l3_N_2_OS (PubChem CID: 50248). Structures were generated by PubChem (National Center for Biotechnology Information,2022).

The physicochemical proprieties of AmpB and TCBZ were assessed to compare their predicted oral bioavailability using Lipinski’s and Veber’s criteria (Veber et al., 2002; Lipinski, 2004). As expected, AmpB presented higher violations than TCBZ (**Table 1**). This is in line with the high level of toxicity that has been described for it, even when using the intravenous route (Laniado-Laborín and Cabrales-Vargas, 2009). AmpB and TCBZ chemical structures are represented in **Figures 1A, B**, respectively.

**Table 1:** Molecular properties of Amphotericin B (AmpB) and Triclabendazole (TCBZ) on Lipinski’s and Veber’s criteria, the number of pan assay interference compounds according to SwissADME web tool, and the presence of drug interactions according to DrugBank.

	AmpB	TCBZ	Limits
**Dug interactions**	None		
**MW**	V	359.66 g/mol	≤ 500
**Log P_o/w_**	-0.39	4.92	≤ 5
**RB**	3	3	≤ 10
**H-Acc**	18	2	≤ 10
**H-Don**	12	1	≤ 5
**tPSA (Å²)**	319.61	63.21 Å²	≤ 140
**PAINS**	0	0	-
**Violations**	4	0	-

MW, molecular weight; Log Po/w, Log of partition-coefficient (consensus LogP on swissADME); RB, number of rotatable bonds; H-Acc: number of Hydrogen bond acceptor. H-Don: number of Hydrogen bond donor; tPSA: molecular polar surface area; PAINS: pan assay interference compounds.

For both drugs, pan assay interference compounds (PAINS) were not identified by the swissADME web tool (Daina et al., 2017). PAINS are substructural features of chemical compounds that are often related to false-positive results in high-throughput screens because they tend to react nonspecifically with numerous biological targets rather than specifically affecting one desired target (Baell and Holloway, 2010). No drug interactions were found by the DrugBank tool for AmpB and TCBZ, suggesting that concomitant use of the drugs is safe.

### 3.2 TCBZ selectivity index is higher than AmpB for intracellular amastigotes

**Figure 2:**
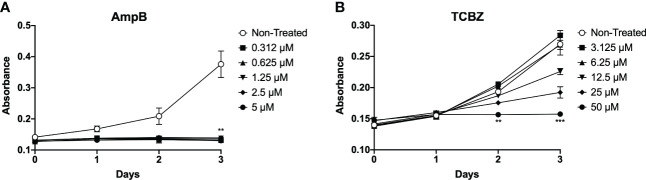
*L. amazonensis* promastigotes proliferation analysis. **(A)** Parasites treated with increasing concentrations of AmpB (0.312, 0.625, 1.25, 2.5 and 5 µM). For AmpB, statistically significant results were presented in all concentrations on the third day (72h) of treatment. **(B)** Parasites treated with increasing concentrations of TCBZ (3.12, 6.25, 12.5, 25 and 50 µM). For TCBZ, statistically significant results obtained in 50 µM concentrations from day 2 (48h) of the analysis. Absorbance (Abs) read at 600 nm. .

To evaluate the effects of TCBZ on parasite growth and determine which treatment time was the best for determining the effects of the drug on promastigote forms, parasites were seeded in 24-well plates, and their growth was estimated by absorbance for three consecutive days. AmpB was used as a comparative drug, as it is one of the most common drugs used to treat leishmaniasis worldwide. The results showed that AmpB affects *L. amazonensis* promastigote proliferation after 72h of treatment for all concentrations tested. On the other hand, TCBZ inhibited parasite growth at higher concentrations after 48h of treatment (**Figure 2**). Thus, promastigote assays were subsequently conducted only at 72h since this time point showed a statistically significant effect for both drugs.

The IC_50_ of TCBZ and AmpB for promastigotes were determined after 72h of treatment by MTT assay. TCBZ IC_50_ was 6 µM, while AmpB IC_50_ was 0.06 µM (**Table 2**).

**Table 2:** Half-maximum cytotoxicity concentration (CC_50_) in macrophages, half-maximum inhibitory concentration (IC_50_) in amastigotes from *L. amazonensis*, and Selectivity Index (SI, CC_50_/IC_50_) of AmpB and TCBZ.

	Macrophages	Amastigotes	
	CC_50_ 48h	IC_50_ 48h	SI 48h
**Triclabendazole**	276.6 ± 47	45.67 ± 7.69	6
**Amphotericin B**	8.1 ± 3.8	2.02 ± 0.26	4

CC_50_ and IC_50_ are shown in μM. Standard deviation is shown by ±.

**Figure 3:**
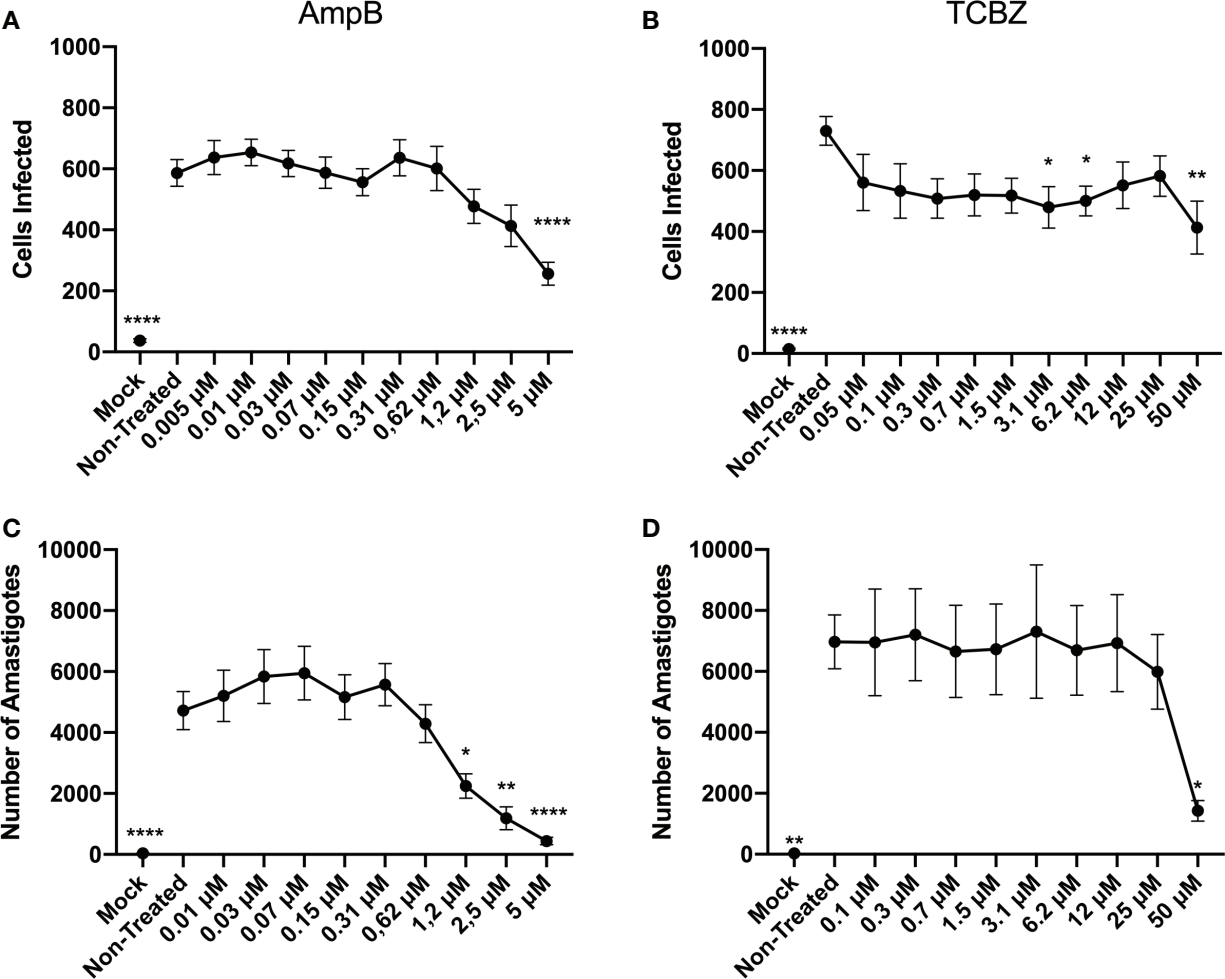
Anti-amastigote effect of AmpB and TCBZ at 48h of treatment. **(A)** Macrophages infected with *L. amazonensis* were treated for 48h in increasing concentrations of AmpB. Statistically significant results can be observed at 5 μM treatment. **(B)** Macrophages infected with *L. amazonensis* were treated for 48h in increasing concentrations of TCBZ. Statistically significant results can be observed at 3.1, 6.2, and 50 μM treatment. Mock cells (without parasite infection) were used as negative control in the Operetta System analysis. **(C)** Number of amastigotes in infected cells treated with AmpB for 48h. Statistically significant results can be observed from 1.2 μM. **(D)** Number of amastigotes in infected cells treated with TCBZ for 48h. Statistically significant results can be observed at 50 μM. * = p < 0.0332, ** = p < 0.0021, **** = p < 0.0001.

Exploratory infection assays were performed for macrophages derived from THP-1 cells infected with intracellular amastigotes. Based on the results obtained (**Supplementary Figure 1**), it was decided that 48h would be the most accurate time-point for amastigote forms, since it presented a higher infection rate then 72h. Although 24h showed similar results when compared to 48h, based on the lack of statistically significant activity of AmpB and TCBZ in promastigotes proliferation analysis (**Figure 2**) this time-point was not considered for further evaluations. Infected macrophages were therefore treated for 48h with growing concentrations of AmpB and TCBZ, and the number of infected cells was analyzed using the Operetta High Content Imaging System. AmpB showed significant results at 5 µM and TCBZ at 3.1, 6.2, and 50 µM (**Figure 3**). The number of total amastigotes was used to determine the IC_50_ for both AmpB (2.02 µM) and TCBZ (45.67 µM).

**Figure 4:**
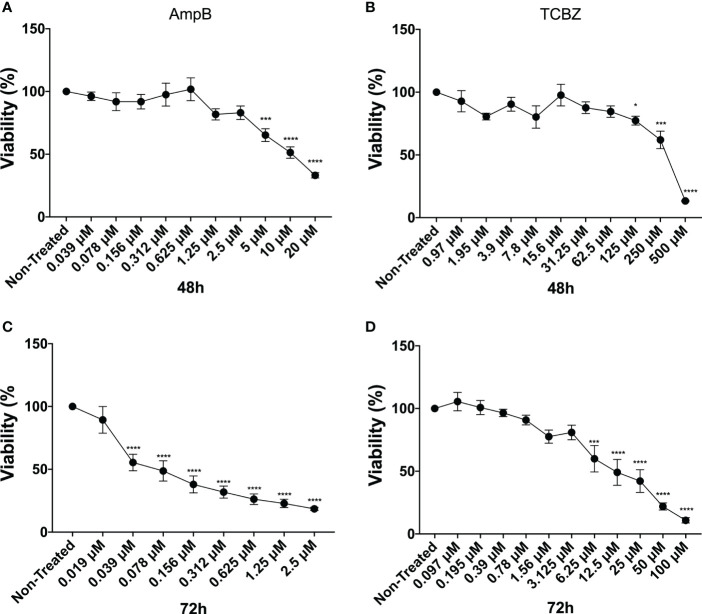
Cytotoxicity assay. Macrophages were treated with increasing concentrations of AmpB and TCBZ. Cytotoxicity evaluated by MTT assay after 48h and 72h of treatment with AmpB **(A)** and **(C)**, respectively. Cytotoxicity evaluated by MTT assay after 48h and 72h of treatment with TCBZ **(B)** and **(D)**, respectively. * = p < 0.0332, *** = p < 0.0002, **** = p < 0.0001.

Half-minimal cytotoxicity concentrations (CC_50_) of TCBZ and AmpB were also determined by MTT assay in THP-1 derivate macrophages. Assays were conducted at 48 and 72h since these were the times evaluated in amastigote and promastigotes assays, respectively. TCBZ showed lower cytotoxicity than AmpB for both 48h (276.6 µM and 8.1 µM, respectively) and 72h (39.4 µM and 0.3 µM, respectively) (**Figure 4**). The selectivity index (SI) was determinate using CC_50_ and IC_50_ of amastigote forms being 4 and 6 for AmpB and TCBZ , respectively (**Table 2**).

### 3.3 Ultrastructural and morphological analysis of treated parasites

**Figure 5:**
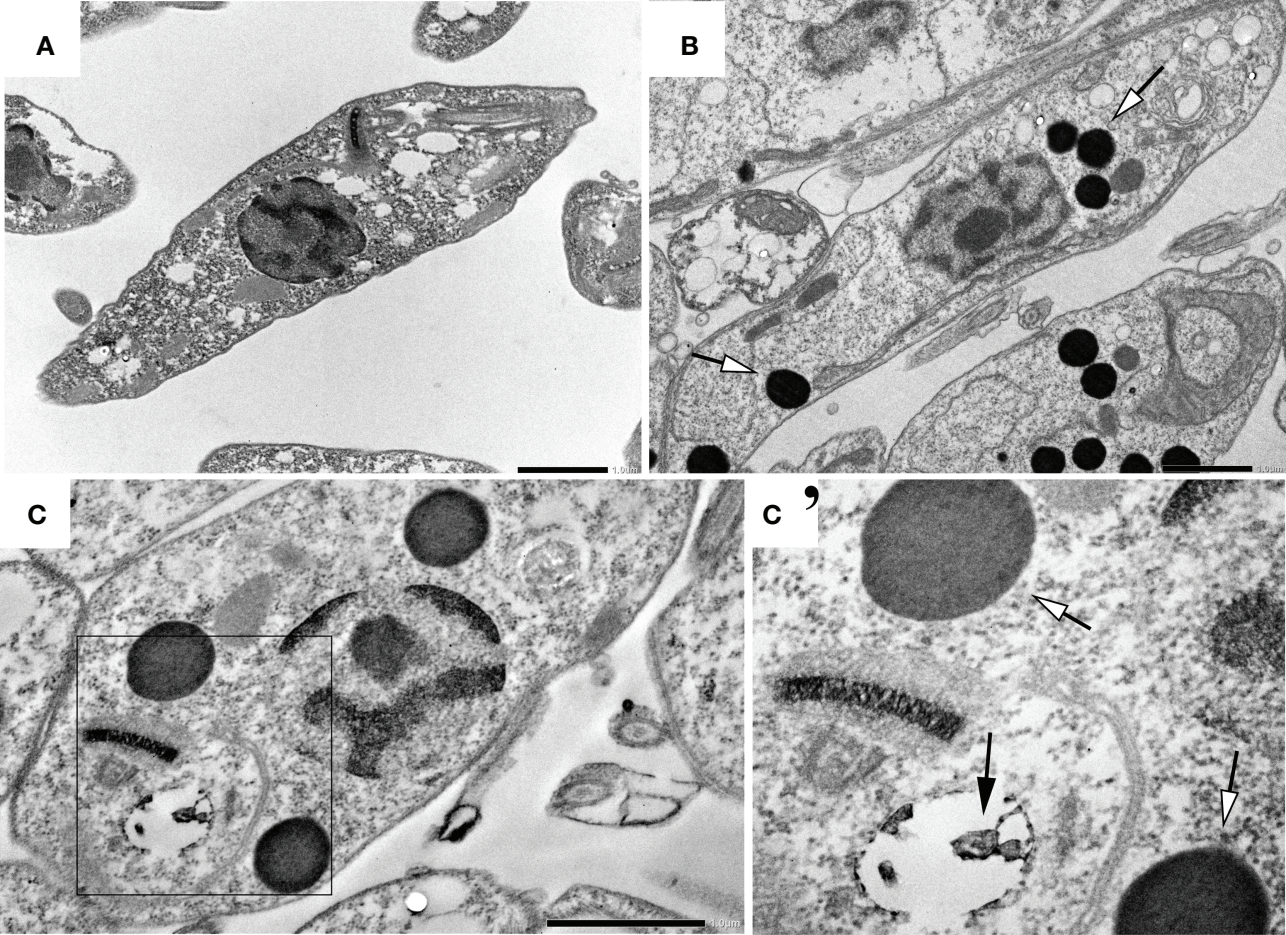
Ultrastructural analysis of *L. amazonensis* promastigotes treated with AmpB and TCBZ. **(A)** Non-treated parasites. **(B)** Parasites treated with AmpB IC_50_ showing large electrondense vesicles (pointed by white arrowheads). **(C)** Parasites treated with TCBZ IC_50_. (C’) Enlargement of the demarked area of **Figure 3C** where treated cells seem not only to present large electron-dense vesicles (white arrowheads) but also vesicles at the flagellar pocket, which were not found in control parasites (black arrows). Bars: 1 µm.

**Figure 6:**
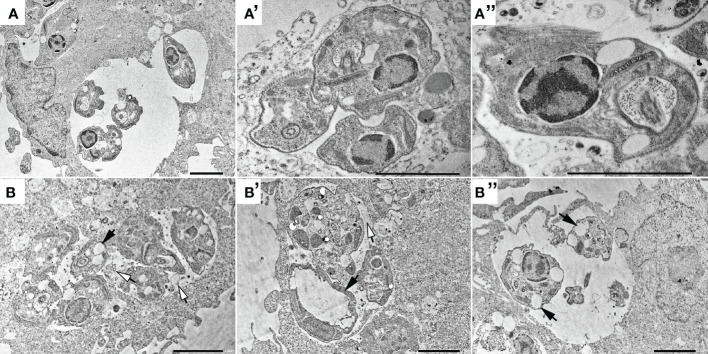
Transmission electron microscopy of non-treated intracellular amastigotes (**A**, **A’** and **A’’**) and treated with 50 μM of TCBZ (**B**, **B’** and **B’’**). Black arrows point to several large vesicles in treated parasites, both in AmpB and TCBZ treatment, absent in control. Also, treated samples seems to present possible cellular debris inside the parasitophorous vacuole (white arrowheads), indicating parasite death. Bars: 2 μm.

Treated parasites (IC_50_ for 72h) were observed by electron microscopy to analyze ultrastructural (**Figure 5**) and morphological (**Figure 6**) alterations induced by TCBZ or AmpB. In *L. amazonensis* promastigotes treated with AmpB IC_50_, several cytoplasmatic electron-dense spherical bodies were observed, which probably contain lipids (**Figure 5B**). TCBZ IC_50_ treated parasites showed the same cytoplasmatic electron-dense spherical bodies observed after AmpB treatment (white arrowheads), and other ultrastructural alterations such as vesicles at the flagellar pocket were also frequently noticed (black arrows, **Figures 5C, C’**). Infected cells were also treated with the highest statistically relevant concentration of TCBZ (50 μM for 48h) to assess the drug’s effects. Although the same electron-dense structures seen in promastigotes were not observed, treated parasites showed intense vesiculation, a feature not seen in untreated parasites (**Figure 6B**). Moreover, structures resembling cell debris inside the parasitophorous vacuole of treated samples were frequently observed in these cells (**Figure 6B-B’’**, white arrowheads).

**Figure 7:**
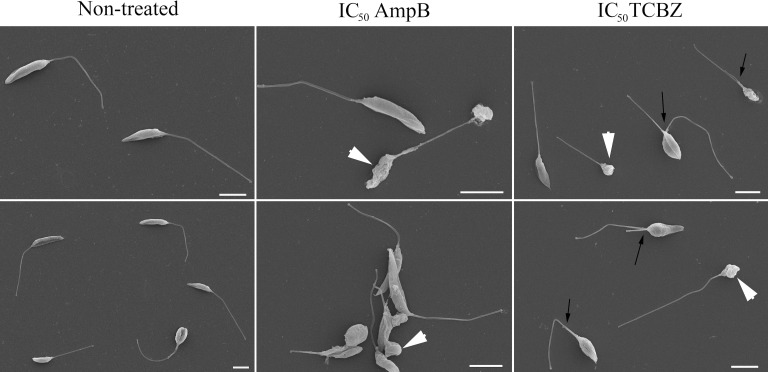
Morphological analysis of *L. amazonensis* promastigotes by scanning electron microscopy (SEM). The first column represents non-treated parasites, and the second and third columns represent parasites treated with AmpB IC_50_/72h and TCBZ IC_50_/72h, respectively. White arrowheads show shrinkage and roundness in the parasites that are presented in both AmpB and TCBZ treatment. Black arrows point to parasites in division, which was observed in a higher frequency in TCBZ treatment, when compared to control or AmpB treatment. Bars: 5 µm.

The morphology of treated promastigotes was observed by SEM. Untreated parasites were used as control and showed normal morphology (**Figure 7**, row 1). AmpB-treated parasites showed morphological alterations such as shrinkage and roundness of the parasite cell body (**Figure 7**, row 2, white arrowheads). *Leishmania amazonensis* promastigotes treated with TCBZ IC_50_ for 72h also showed morphological alterations like shrinkage and roundness in the parasite shape cell (**Figure 7**, row 3, white arrowheads). Moreover, dividing cells were frequently observed, indicating a possible alteration in the cytokinesis process (**Figure 7**, row 3, black arrows).

### 3.4 Cell cycle is affected by TCBZ treatment

**Figure 8:**
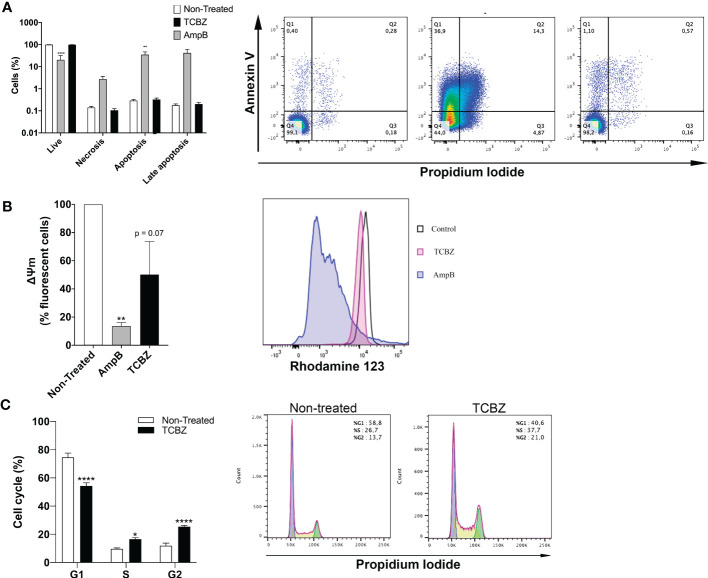
Flow cytometer assays in promastigotes treated with IC50 of AmpB and TCBZ for 72h. **(A)** Cell death evaluation with co-staining of Annexin V and PI of non-treated and TCBZ treated parasites. Typical dot plots of one representative experiment are shown. Q1: apoptosis, Q2: late apoptosis. Q3: living cells. Q4: necrosis. **(B)** Membrane potential evaluated with Rod123 assay. Typical histograms of one representative experiment are shown. **(C)** Cell cycle evaluation with PI staining. Typical histograms of one representative experiment are shown. Values are represented in percentage (%) of 10.000 evaluated events. * = p < 0.0332, ** = p < 0.0021, **** = p < 0.0001.

To better understand TCBZ’s mechanism of action, treated parasites were analyzed by flow cytometry for cell death, cell cycle, and mitochondrial membrane potential. Cell death analysis showed that treatment with AmpB increased the number of stained parasites using both Annexin V and PI (**Figure 8A**). Untreated promastigotes showed 99.4% of living cells, 0.29% of apoptosis, 0.14% of necrosis, and 0.17% of late apoptosis. Similarly, TCBZ-treated parasites showed 99.4% of living cells, 0.3% of apoptosis, 0.1% of necrosis, and 0.2% of late apoptosis. On the other hand, AmpB-treated parasites showed 20.4% of living cells, 34.9% of apoptosis, 2.7% of necrosis, and 42% of late apoptosis.

Mitochondrial membrane potential was evaluated in treated promastigotes. Parasites treated with AmpB showed a clear loss of fluorescence when compared to controls (93%), while TCBZ-treated parasites showed a decrease of 50% in fluorescence (p = 0.07, **Figure 8B**).

The cell cycle was analyzed with PI staining, and non-treated parasites were used as control. TCBZ-treated *L. amazonensis* promastigotes showed significant alterations in cell cycle phases, with a decrease in G1 from 74.6% to 54.3% and an increase in S and G2 from 9.7% to 16.6% and 11.9% to 25.4%, respectively (**Figure 8C**).

### 3.5 ROS evaluation and lipid quantification

**Figure 9:**
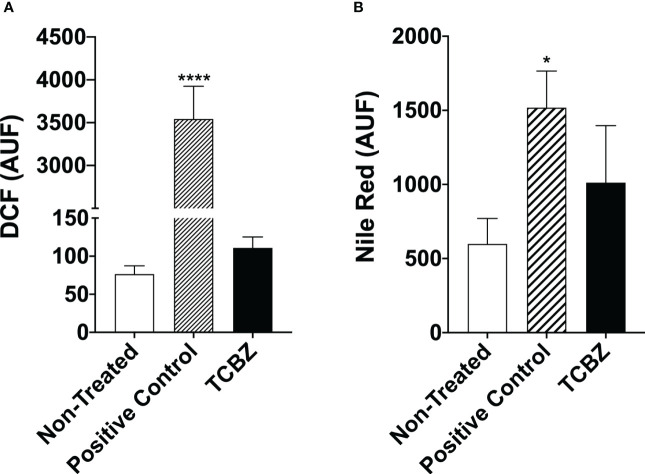
Reactive oxygen species evaluation and lipid quantification in *L. amazonensis* promastigotes treated with TCBZ. **(A)** ROS of *L. amazonensis* promastigotes treated with TCBZ IC_50_ for 72h. **(B)** Lipid quantification of *L. amazonensis* promastigotes treated with TCBZ IC_50_ for 72h. AUF – arbitrary units of fluorescence. * = p < 0.0332, ****= p < 0.0001.

ROS were quantified to access cell death pathway. For this, promastigote forms were treated with IC_50_ of both drugs for 72h. Results showed that treated cells present a ROS profile that is similar to non-treated parasites, indicating that the anti-*Leishmania* effect of TCBZ may not be using the ROS production pathway (**Figure 9A**).

Furthermore, to verify if electron-dense vesicles observed by TEM could be filled with neutral lipids, this lipid class was quantified in promastigotes after 72h of treatment with IC_50_. We found a 69% tendency to increase the fluorescence intensity of parasites treated with TCZB compared to untreated promastigotes. However, no statistical difference was found (**Figure 9B**).

### 3.6 Synergistic effect of TCBZ

The next step was to examine whether TCBZ and AmpB have a synergistic effect that lowers the IC_50_ of AmpB and, consequently, its toxicity, elevating the SI. In order to do this, combined proportions of 10:1, 5:1, 4:1, and 1:1 concentrations for TCBZ and AmpB were prepared, and infected macrophages were treated for 48h. These ratios were chosen based on the previous observations that TCBZ alone appears to have a comparable anti-*Leishmania* effect in doses 10x higher than AmpB. Also, as the IC_50_ of AmpB is around 2 μM, the focus of this assay was to evaluate if TCBZ was capable of lowering AmpB’s IC_50_ through a synergistic effect. The results showed that at all ratios tested, the IC_50_ of the drug in combination ranged from three to 120 times lower than the drugs alone (**Table 3**). Cytotoxicity assay was also performed in macrophages derived from THP-1 cells. For 1:1 ratio, CC_50_ was 29.34 μM for both drugs. For 1:4, 1:5 and 1:10 ratios, AmpB CC_50_ was 14.73, 27.77 and 34.16 μM, respectively, while TCBZ CC_50_ was 33.60, 36.14 and 21.55 μM, respectively. Regarding SI, AmpB and TCBZ presented the value of 77.2 for 1:1 ratio. For ratios of 1:4, 1:5 and 1:10, AmpB presented values of 39.8, 74.4 and 69.7, respectively, while TCBZ presented values of 46.6, 15.7 and 5.5, respectively.

**Table 3:** IC_50_ of intracellular amastigote forms in each proportion analyzed (1:1, 4:1, 5:1, 10:1 for TCBZ and AmpB, respectively).

Proportion	IC_50_ TCBZ (μM)	IC_50_ AmpB (μM)	FICs TCBZ	FICs AmpB	ΣFICs
**01:01**	0.38	0.38	0.01	0.19	0.20
**04:01**	0.72	0.37	0.02	0.19	0.20
**05:01**	2.30	0.40	0.05	0.20	0.25
**10:01**	4.97	0.49	0.11	0.25	0.36
**00:01**	**45.67**	**2.02**		**χΣ FICS**	**0.25**

0:1 proportion refers to the IC_50_ of each drug alone. All experiments were made in biological replicate; numbers represent average with R squared > 0.9. χΣFICS mean the average of the sum of all ΣFICs proportions.

**Figure 10:**
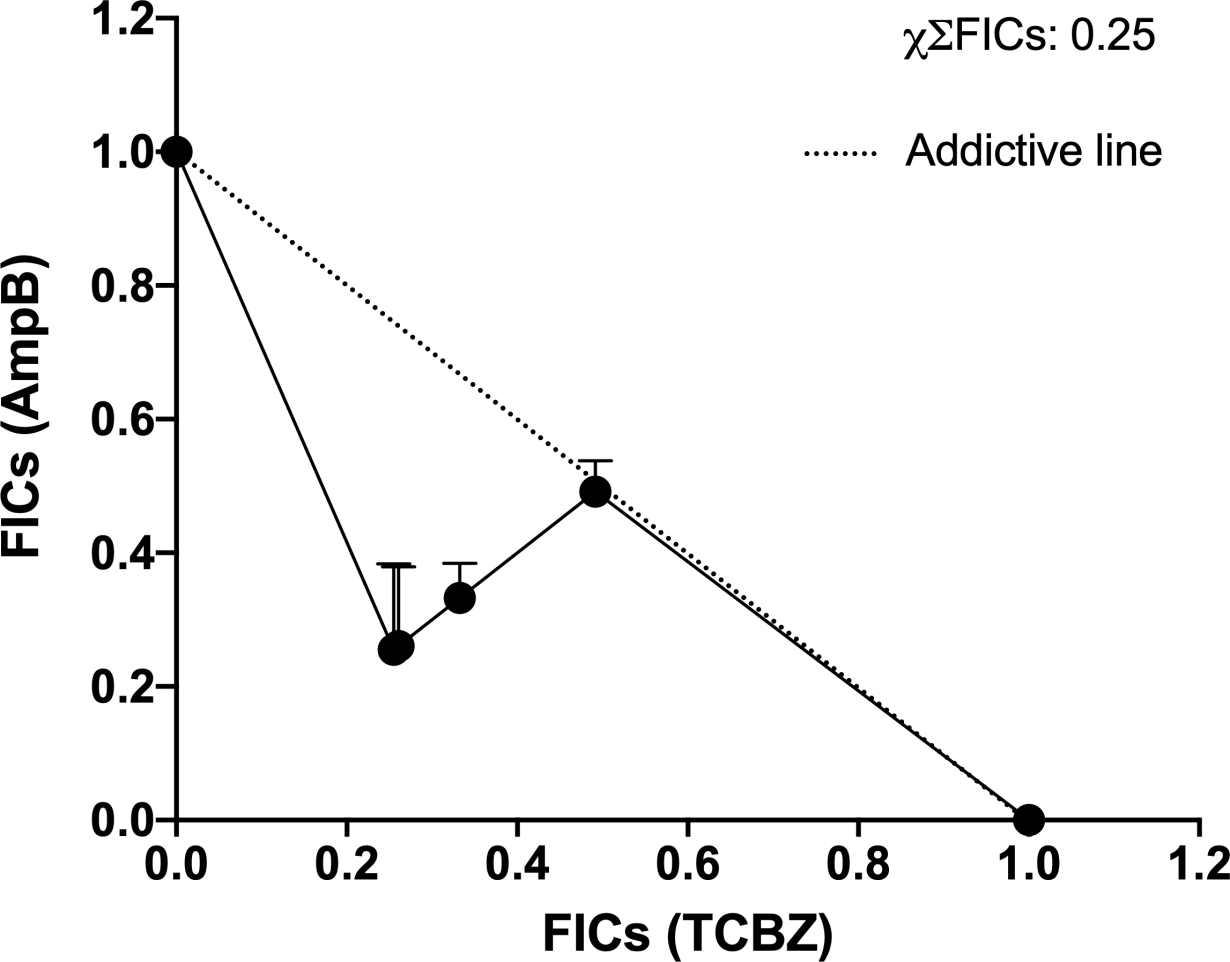
Isobologram of ΣFICs of TCBZ and AmpB. Each point represents FICs of each proportion (10:1, 5:1, 4:1, 1:1 for TCBZ and AmpB, respectively). The dotted line represents the addictive line. The result of χΣFICs of all ratios analyzed is at the upper right.

Once the IC_50_ for each drug in each combination was determined, the ΣFICs equation (see item 2e) was used to calculate the average of the sum FICs (χΣFICs). We found that the combination of TCBZ and AmpB resulted in a synergistic effect with a χΣFICs of 0.25. According to Odds (2003), the drug combination is considered synergic when χΣFICs ≤ 0.5. An isobologram plotted with every analyzed ratio also demonstrated FICs below the addictive line that represents theoretical addictive (or non-interactive) χΣFICs 1 value, corroborating with χΣFICs value found in this study (**Figure 10**).

## 4 Discussion

Treatment for leishmaniasis has changed very little since the early 90s. This is not because available treatments are highly efficient. Rather, it is probably due to a very low level of investment in this neglected disease. We are far from having a vaccine for leishmaniasis and lack a less toxic, cheaper, and more effective drug. Drug repositioning may potentially be used to save time and money in this situation. In this study, we present the results of a benzimidazole derivate that could be a prospective treatment for leishmaniasis. Firstly, promastigote growth was evaluated in the presence of TCBZ and AmpB for 72h in increasing drug concentrations to determine IC_50_. Intracellular amastigotes were treated for 48h for the same purpose. Regarding macrophage cytotoxicity, although tested concentrations were 10 times higher for TCBZ than for AmpB, CC_50_ values were comparable for both drugs. Furthermore, both drugs had similar SI for *L. amazonensis* amastigotes.

Ultrastructural analysis by transmission electron microscopy showed that treated parasites exhibited not only electron-dense structures compatible with lipid accumulation but also an alteration in flagellar pocket size, with the presence of small electron-dense vesicles. These alterations have already been shown in studies that tested possible sterol metabolism inhibitors in *L. amazonensis*, *L. chagasi*, and *L. braziliensis* (Rodrigues et al., 2008; Oliveira et al., 2009; Ceole et al., 2017). The inference about electrodense structures can be explained by osmium tetroxide post-fixation, which has a high lipid affinity and corroborates with sterol biosynthesis target drugs (de Souza and Rodrigues, 2009). So far, the mechanism of action of benzimidazole derivate drugs has only shown microtubule and protein inhibition in a variety of parasites (Robinson et al., 2002). To our knowledge, this is the first time TCBZ has shown any relation to lipid or sterol pathways.

Although cellular death and mitochondrial membrane potential analysis did not show substantial results in TCBZ-treated promastigotes, the cell cycle showed significant modification, with an increase in the number of parasites in the S and G2 phases. Indeed, it has been shown that some drugs that target the sterol biosynthesis pathway can alter the cell cycle, probably due to changes in the availability of essential sterols (Oliveira et al., 2009; Almeida-Souza et al., 2016), in line with TEM results. Another possible explanation is that it is a consequence of alterations in cytoskeleton organization due to direct influence on microtubule polymerization (Robinson et al., 2002) or nuclear membrane alteration, preventing complete cytokinesis (de Souza and Rodrigues, 2009). In addition, several cells in division were found in SEM when treated with TCBZ, in accordance with cell cycle results since cytokinesis appears to be affected. Nevertheless, the quantification of ROS and neutral lipids did not show significant results.

When taken together, the results point to a possible anti-*Leishmania* effect of TCBZ, suggesting that this drug could be an interesting candidate to complement anti-leishmania therapy. Additionally, TCBZ may not only use microtubules as a pharmacological target but also interfere in lipid metabolism.

Since TCBZ showed a possible anti-*Leishmania* effect, we hypothesized that this drug could have a synergistic effect when combined with the anti-*Leishmania* effect of AmpB. So far, one of the few compounds containing imidazole in its composition tested in combination with AmpB for leishmaniasis treatment did not have a synergistic statistically significant effect (Rodriguez et al., 1995). However, because TCBZ has already shown successful repurposing as a bactericidal (Pi et al., 2021) and as a fasciolosis treatment in sheep, when used combined with other drugs, even in resistant parasites (Tabari et al., 2022), this specific combination may lead to desirable results. AmpB has already been used in successful treatments in combination with miltefosine, meglumine antimoniate, and pentamidine (Goswami et al., 2020; Ramesh et al., 2020; Vechi et al., 2020). Indeed, we found that TCBZ and AmpB have synergic effects *in vitro* when administrated in combination, and no interaction between these two drugs was found by the DrugBank prediction *in silico*, indicating that it is safe to combine them.

Patients often abandon treatment with AmpB due to its side effects and the fact that several trips to the hospital are required for parenteral administration (Neves et al., 2011). However, other ways to administer AmpB, such as through aerosol in local applications and oral administration, have already shown good results in combination with other drugs and the diminution of side effects (Basile et al., 2020; Parvez et al., 2020). The option of oral administration of AmpB added to the fact that TCBZ has demonstrated not only synergic effect when combined with AmpB, but it is also already an approved medication for oral administration, showing that this drug combination could be a prospective alternative treatment for leishmaniasis.

In summary, TCBZ appears to have an anti-*Leishmania in vitro* effect against *L. amazonensis* as it shows a decrease in parasite growth, cell cycle arrest in the S and G2 phases, and morphologically bigger cell body for several parasites in cell division. Also, TCBZ and AmpB have shown synergic effects on intracellular amastigotes when studied in combination. Although additional work is needed, including *in vivo* assays, these results show that TCBZ displays promising *in vitro* anti-*Leishmania* activity, especially in combination with AmpB, with the attractive characteristic that TCBZ would be a repurposed drug available in oral form.

## Data availability statement

The original contributions presented in the study are included in the article/**Supplementary Material**. Further inquiries can be directed to the corresponding author.

## Author contributions

BB designed the study, performed experiments, analyzed the data, and wrote and prepared the manuscript. GB analyzed the data. FT-P performed experiments and analyzed the data. FF wrote and prepared the manuscript. LSM designed the study, analyzed the data, and wrote and prepared the manuscript. All authors contributed to the article and approved the submitted version.
