# Supplementary material for: In vitro anti-Leishmania activity of triclabendazole and its synergic effect with amphotericin B
Source: Front Cell Infect Microbiol. 2023 Jan 9;12:1044665. doi: 10.3389/fcimb.2022.1044665 (PMC9868945; doi:10.3389/fcimb.2022.1044665)
Supplement: Supplementary file 1 [file DataSheet_1.docx]

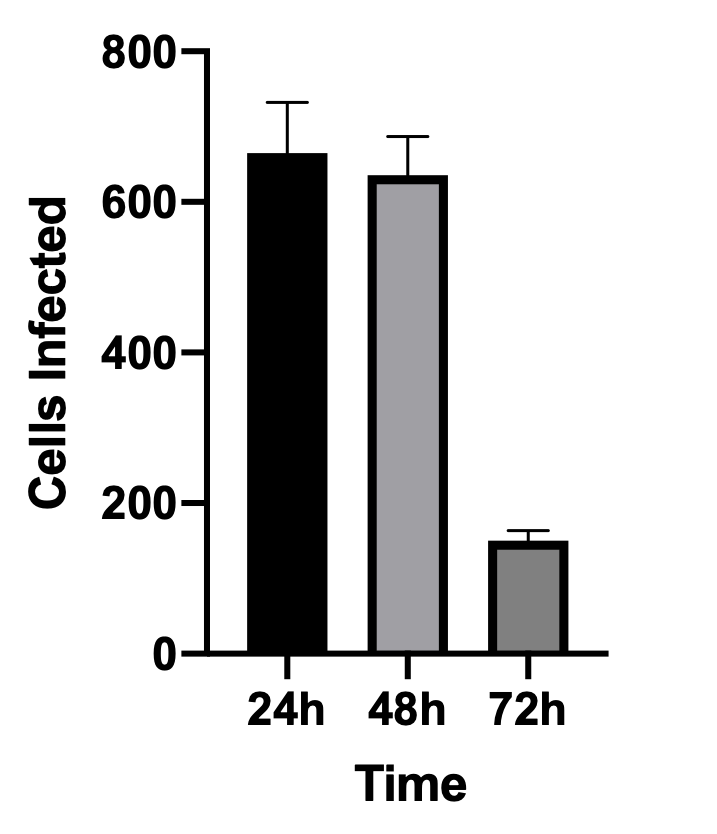


**Supplementary Figure 1. Infection of *L. amazonensis* in macrophages.** Cells were maintained without treatment for 24, 48 and 72h. While 72h show a significantly reduction of infection probably due to cell lysis, 24 and 48h show a high rate of infection (>600 macrophages out of 3 analyzed wells). Exploratory infection was made in technical triplicate.
